# Performance characteristics between TDx®FLx and TBA™-25FR for the therapeutic drug monitoring of methotrexate

**DOI:** 10.1186/s40780-016-0042-y

**Published:** 2016-03-08

**Authors:** Tetsuya Kaneko, Takashi Fujioka, Yosuke Suzuki, Yuhki Sato, Hiroki Itoh

**Affiliations:** Department of Clinical Pharmacy, Oita University Hospital, Hasama-machi, Oita 879-5593 Japan

**Keywords:** Methotrexate, High-dose Methotrexate, Therapeutic drug monitoring, Automatic immunoassay systems

## Abstract

**Background:**

High-dose methotrexate (HDMTX) is used in the treatment of certain malignancies, including leptomeningeal metastases, systemic non-Hodgkin lymphoma, acute lymphoblastic leukemia, and osteosarcoma. High circulating levels of methotrexate can cause severe myelosuppression. The present study aimed to examine the differences in plasma MTX concentrations measured by two immunoassay systems currently available in the Japanese market, a TD_X_/FL_X_ analyzer and a TBA-25FR analyzer.

**Methods:**

A total of 69 plasma samples from 16 patients were assayed by a fluorescence polarization immunoassay technique using a TDx/FLx analyzer (Abbott Diagnostics, Chicago, Illinois, U.S.A.) and a homogeneous enzyme immunoassay technique using a TBA-25FR analyzer (Toshiba Medical Systems, Tokyo, Japan).

**Results:**

Assay results were very consistent between the two systems, with good correlation 24 h after the start of treatment (TBA-25FR = 1.06・TD_X_/FL_X_, −1.31, *r* = 0.99), 48 h after the start of treatment (TBA-25FR = 1.00・TD_X_/FL_X_, +0.027, *r* > 0.99), and 72 h after the start of treatment (TBA-25FR = 1.09・TD_X_/FL_X_, +0.011, *r* > 0.99).

**Conclusions:**

The calibration curve spanned one order of magnitude with a linear working range from the lowest to the highest standard. The standard deviations show the excellent reproducibility of repeated measurements at each standard level for both immunoassay systems. However, when using the TBA-25FR, it is necessary to perform measurements in the low-concentration range with care.

## Background

Methotrexate (MTX; (2S)-2-[[4-[(2,4-diaminopteridin-6-yl)methylmethylamino] benzoyl] amino] pentanedioic acid)) is an antimetabolite drug that acts by inhibiting dihydrofolate reductase, disrupting purine synthesis, and preventing cell division. This results in the depletion of intracellular pools of reduced folates required for DNA synthesis. Rapidly dividing malignant cells that require greater amounts of reduced folates are preferentially affected by MTX, resulting in the cessation of DNA synthesis and eventual cell death. High-dose MTX (HDMTX) is used in the treatment of certain malignancies, including leptomeningeal metastases, systemic non-Hodgkin lymphoma, acute lymphoblastic leukemia, and osteosarcoma [1]. Methotrexate is predominantly eliminated by the kidneys [2–4]. Indeed, 70–90 % of the administered MTX dose is excreted unchanged in the urine [3]. Despite the fact that HDMTX is a very effective treatment, plasma MTX concentrations may become excessively high in a small proportion of patients, resulting in toxicity and elimination delay [5]. High circulating levels of methotrexate can cause severe myelosuppression. Wide inter-individual variability has been reported for the pharmacokinetic profiles of MTX in cases treated with HDMTX therapy [6]. The plasma concentration of MTX at 24 h can predict efficacy, whereas the plasma concentrations at 48 and 72 h can reflect the excretion of MTX. Elimination delay is indicated by MTX concentrations > 1.0 μmol/L at 48 h and > 0.1 μmol/L at 72 h [7, 8]. Therefore, frequent determination of MTX concentrations is needed to safely manage individual patients receiving HDMTX therapy [9, 10]. This determination is usually carried out by automated immunoassay because urgent analysis may be required. A TD_X_/FL_X_ analyzer had been used but we were changed to TBA-25FR analyzer for discontinued measuring reagent for use in a TD_X_/FL_X_ analyzer. There is a possibility that results of blood concentrations in same sample measured by the different assay systems may differ by those measurement equipment. However, information about the correlation of the measured values of MTX between TD_X_/FL_X_ analyzer and TBA-25FR analyzer is small.

The present study aimed to examine differences in plasma MTX concentrations measured by two immunoassay systems currently available in the Japanese market. We compared the plasma MTX concentrations measured by a TD_X_/FL_X_ analyzer and a TBA-25FR analyzer.

## Methods

### Patients

Medical records were reviewed to identify hospitalized Japanese patients treated with methotrexate at Oita University Hospital between May 2013 and December 2013. Patients who received HDMTX therapy for certain malignancies including leptomeningeal metastases, systemic non-Hodgkin lymphoma, acute lymphoblastic leukemia, and osteosarcoma were included. This study was approved by the Ethics Committee of Oita University. Since blood samples were collected for therapeutic drug monitoring and laboratory testing as part of routine patient care, written informed consent was not necessary.

### Automatic immunoassay systems

The plasma MTX concentrations were determined by a fluorescence polarization immunoassay technique using a TD_X_/FL_X_ analyzer (Abbott Diagnostics, Chicago, Illinois, U.S.A.) and a homogeneous enzyme immunoassay technique using a TBA-25FR analyzer (Toshiba Medical Systems, Tokyo, Japan). For the measurement of the a TDX/FLX analyzer and a TBA-25FR analyzer we were using the TDx-Methotrexate Dynapac. II (Abbott Diagnostics, Chicago, Illinois, U.S.A. Lot No.: 35351 M500) and Nanopia eTDM Methotrexate (SEKISUI MEDICAL CO., LTD. Tokyo, Japan. Lot No.: 804REL). Assays were performed at Oita University Hospital according to manufacturer instructions.

### Measurement of plasma methotrexate concentrations

Plasma MTX concentrations were measured by TD_X_/FL_X_ and TBA-25FR at 24, 48, and 72 h after the start of treatment. According to manufacturer’s information for the TD_X_/FL_X_ analyzer, the lower limit of quantification of the assay is 0.02 μmol/L. Blood samples exceeding the upper limit of the calibration range (0.00–1.00 μmol/L) were diluted according to the manufacturer’s protocol. According to manufacturer’s information for the TDx-Methotrexate Dynapac. II, the intra- and inter-day accuracy were lower than 115 % at the 0.07, 0.40, 0.80, 5, 50 and 500 μmol/L concentration. And coefficient of variation values were less than 15 %. According to manufacturer’s information for the TBA-25FR analyzer, the lower limit of quantification of the assay was 0.04 μmol/L. Blood samples exceeding the upper limit of the calibration range (0.00–1.20 μmol/L) were diluted according to the manufacturer’s protocol. According to manufacturer’s information for the Nanopia eTDM Methotrexate, the intra- and inter-day accuracy were lower than 115 % at the 0.00, 0.05, 0.15, 0.25, 0.50 and 1.20 μmol/L concentration. And coefficient of variation values were less than 15 %.

### Comparisons between the same samples

They were evaluated for accuracy intra-day, inter-day and interobserver using the same sample. We used 0.07 (CONTROL Low), 0.4 (CONTROL Middle) and 0.8 (CONTROL High) μmol/L of The Methotrexate II Controls (Abbott, Lot 37001 M100) as spiked MTX concentration. This study performed according to the FDA or the PMDA guidance for bioanalytical method validation.

## Findings

### Results

A total of 16 patients (12 men and 4 women) were included in the study. Their median (range) age was 32 (8–73) years and mean body weight was 50.8 (26.6–75.6) kg (Table 1). The number of total samples were 69. The number of samples that were measured at each time point 24, 48, and 72 h after the start of treatment was 23, 18, 18. The number of samples measured at other than 24, 48, and 72 hours after the start of treatment was 10.

**Table 1:** Patient characteristics and clinical laboratory data

Age (years)	32 (8–73)
Gender [male/female]	12/4
Height (cm)	165 (124.4–178)
Body weight (kg)	50.8 (26.6–75.6)
WBC	5.8 (1.3–22.5)
RBC	3.4 (2.8–4.6)
HGB	10.6 (8.3–15.1)
HCT	31.6 (24.1–41.7)
CRP (mg/dL)	0.20 (0.02–1.85)
Alb (g/dL)	3.8 (2.7–4.5)
TP (g/dL)	6.4 (4.7–7.4)
T.Bil (mg/dL)	0.66 (0.19–1.74)
AST (IU/L)	28.2 (14.2–177.7)
ALT (IU/L)	33.8 (12.5–198.4)
ALP	322.0 (189.0–514.0)
γ-GTP	49.4 (11.1–222.8)
BUN (mg/dL)	12.2 (2.9–30.6)
SCr (mg/dL)	0.60 (0.23–2.05)
CCr	99.6 (43.5–219.1)

Data are expressed as median and interquartile range unless otherwise stated

**Fig. 1:**
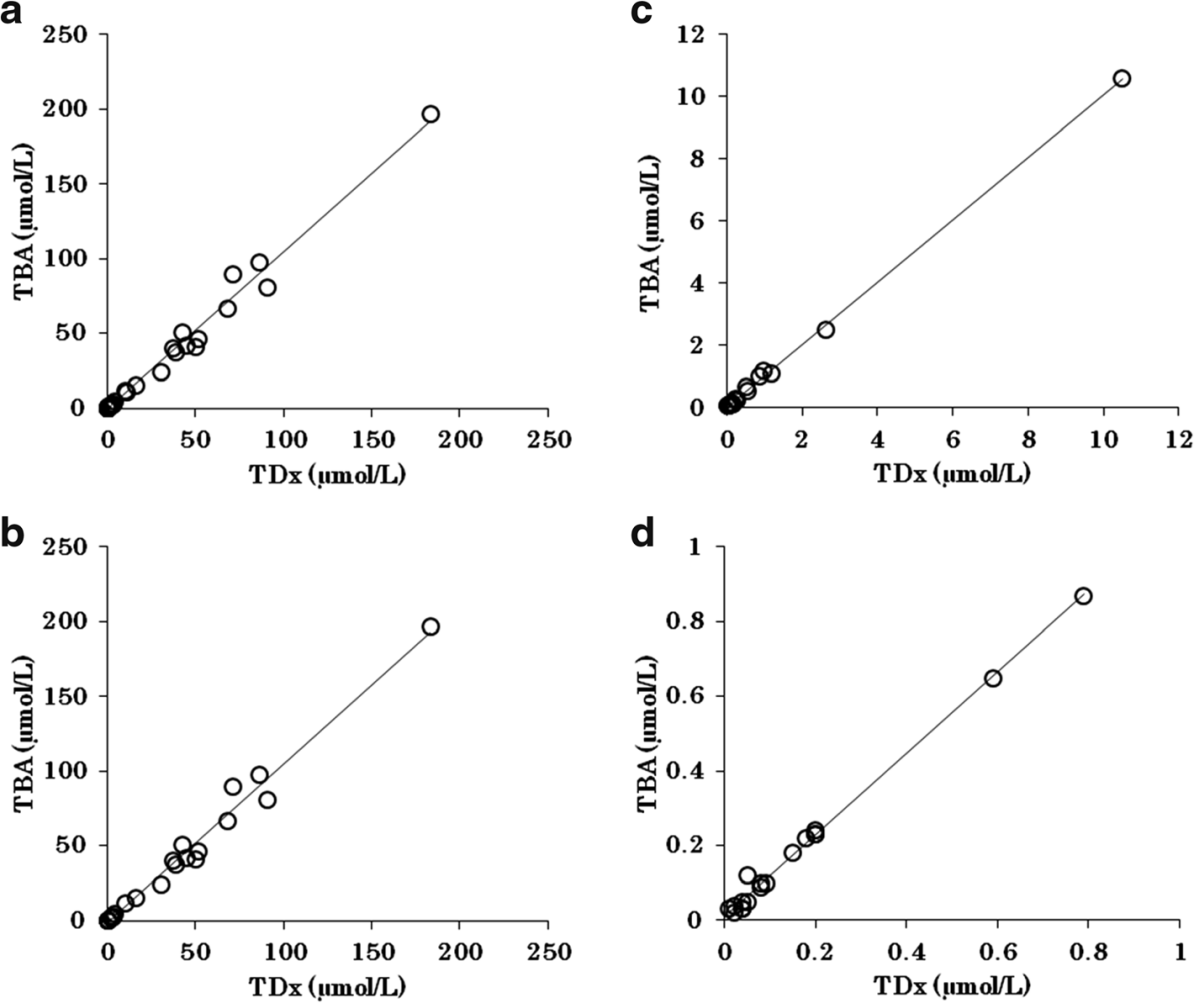
Scatter plots with fits correlating methotrexate results (μmol/L) obtained by the reference method TBA-25FR with results obtained by TD_X_/FL_X_ immunoassay at total samples (**a**), 24 h (**b**), 48 h (**c**), and 72 h (**d**) after the start of treatment. Assay results were very consistent between the two immunoassay systems, with good correlation total samples after the start of treatment (TBA-25FR = 1.05*TD_X_/FL_X_, −0.29, *r* = 0.99; Fig. 1a), 24 h after the start of treatment (TBA-25FR = 1.06*TD_X_/FL_X_, −1.31, *r* = 0.99; Fig. 1b), 48 h after the start of treatment (TBA-25FR = 1.00*TD_X_/FL_X_, +0.027, r > 0.99; Fig. 1c), and 72 h after the start of treatment (TBA-25FR = 1.09*TD_X_/FL_X_, +0.011, r > 0.99; Fig. 1d)

Assay results were very consistent between the two immunoassay systems, with good correlation total samples after the start of treatment (TBA-25FR = 1.05*TD_X_/FL_X_, −0.29, *r* = 0.99; Fig. 1a), 24 h after the start of treatment (TBA-25FR = 1.06*TD_X_/FL_X_, −1.31, *r* = 0.99; Fig. 1b), 48 h after the start of treatment (TBA-25FR = 1.00*TD_X_/FL_X_, +0.027, *r* > 0.99; Fig. 1c), and 72 h after the start of treatment (TBA-25FR = 1.09*TD_X_/FL_X_, +0.011, *r* > 0.99; Fig. 1d). The data for intra- and interday precision and accuracy are presented in Table 2.

**Table 2:** Intra- and interday precision and accuracy

Intraday variation						
Spiked MTX concentration (μmol/L)	TD_X_/FL_X_			TBA-25FR		
	Measured concentration (μmol/L) (mean ± S.D.)	Accuracy (%)	CV (%)	Measured concentration (μmol/L) (mean ± S.D.)	Accuracy (%)	CV (%)
0.07	0.077 ± 0.013	110	16.3	0.096 ± 0.011	137.1	11.2
0.4	0.41 ± 0.025	102.5	6.2	0.41 ± 0.016	102.5	4.0
0.8	0.85 ± 0.041	106.3	4.9	0.92 ± 0.040	115	4.4
Interday variation						
Spiked MTX concentration (μmol/L)	TD_X_/FL_X_			TBA-25FR		
	Measured concentration (μmol/L) (mean ± S.D.)	Accuracy (%)	CV (%)	Measured concentration (μmol/L) (mean ± S.D.)	Accuracy (%)	CV (%)
0.07	0.073 ± 0.010	104.3	14.5	0.96 ± 0.013	137.1	13.1
0.4	0.41 ± 0.018	102.5	4.3	0.43 ± 0.020	107.5	4.6
0.8	0.83 ± 0.045	103.8	5.4	0.93 ± 0.051	116.3	5.4

## Discussion

The time calibration curve showed excellent linearity, with correlation coefficients generally ≥ 0.99. The calibration curve spanned one order of magnitude with a linear working range from the lowest to the highest standard. The standard deviations show the excellent reproducibility of repeated measurements at each standard level. Linear regression of correlation data revealed that the homogeneous enzyme immunoassay technique using a TBA-25FR analyzer produced positive bias compared with the fluorescence polarization immunoassay technique using a TD_X_/FL_X_ analyzer.

In the low-concentration and high-concentration regions, the TBA-25FR showed less variation when compared to TD_X_/FL_X_, but the positive bias needs to be taken into consideration.

The coefficient of variation values were less than 15 % for the TBA-25FR. Results were reproducible, although intra- and inter day accuracy was higher than 130 % at the 0.07 μmol/L concentration. This slight change means that although the variation was low and results were reproducible, the 130 % accuracy at the 0.07 concentration is an indication of less accuracy at this concentration (or a positive bias, as mentioned above) (Table 2). Interobserver precision and accuracy are result similar to the intra- and inter day accuracy (Table 3). Cause of the results it was thought to be due to the use of TDx’s QC sample. Because I perform the examination of the accuracy intra-day, inter-day and interobserver in this study in TDx-Methotrexate Dynapac. II which is QC for TDx, it is expected that TDx turned out good in comparison with TBA. The need that I evaluated using a standard reagent from this result became clear. TBA-25FR methotrexate assays have inherent limitations at the 0.07 μmol/L and 0.8 μmol/L spiked MTX concentrations, giving higher measured concentrations compared to the TD_X_/FL_X_. At the 0.07 μmol/L concentration, the accuracy of TBA-25FR immunoassay was lower than immunoassay by TD_X_/FL_X_. The cause is thought to be that the TBA-25FR analyzer has a higher lower limit of quantification concentration than the TD_X_/FL_X_. Because an actual value becomes higher than a theoretical value in the vicinity of detection limit of 0.04 g/mL in A, in A, it is thought that it is with a high value than B in the low-concentrated neighborhood.

**Table 3:** Interobsever precision and accuracy

Interobsever (*n* = 10)						
Spiked MTX concentration (μmol/L)	TD_X_/FL_X_			TBA-25FR		
	Measured concentration (μmol/L) (mean ± S.D.)	Accuracy (%)	CV (%)	Measured concentration (μmol/L) (mean ± S.D.)	Accuracy (%)	CV (%)
0.07	0.067 ± 0.005	95.7	7.9	0.103 ± 0.012	147.1	11.6
0.4	0.40 ± 0.021	100	5.3	0.43 ± 0.021	107.5	4.8
0.8	0.80 ± 0.052	100	6.4	0.96 ± 0.053	120	5.5

## Conclusions

The TBA-25FR immunoassay system can play a valuable diagnostic role in patient care as a valuable adjunct to therapeutic and clinical treatment. It is able to distinguish underdosing from overdosing in HDMTX therapy. However, when using the TBA-25FR, it is necessary to perform measurements in the low-concentration range with care.
